# Computed tomography-based body composition is associated with adverse clinical outcomes among older patients with sepsis in the emergency department

**DOI:** 10.1007/s41999-023-00756-3

**Published:** 2023-02-13

**Authors:** Qiujing Li, Na Shang, Qian Gao, Li Yang, Shubin Guo

**Affiliations:** 1 Department of Emergency Medicine, Beijing Shijitan Hospital, Capital Medical University, Beijing, China; 2 Department of Emergency Medicine, Capital Medical University of Rehabilitation Medicine, Beijing Bo’Ai Hospital, China Rehabilitation Research Center, Beijing, China; 3 Department of Emergency Medicine, Beijing Key Laboratory of Cardiopulmonary Cerebral Resuscitation, Beijing Chao-Yang Hospital, Capital Medical University, No 8, South Road of Worker’s Stadium, Chaoyang District, Beijing, 100020 China

**Keywords:** Body composition, Sepsis, Computed tomography, Sarcopenia, Older adults

## Abstract

**Purpose:**

To investigate the association between body composition and adverse clinical outcomes in older patients with sepsis in the emergency department.

**Methods:**

Body composition, including the skeletal muscle area, skeletal muscle index (SMI), mean skeletal muscle density (SMD), and intramuscular fat area, was measured at the level of the third lumbar vertebra (L3) on abdominal computed tomography scans. Clinical outcomes included 90-day mortality, 90-day readmission, and discharge to long-term care. According to sex-specific cut-off values of L3 SMI and SMD, patients were divided into low SMI, low SMD, both low SMI and low SMD, and neither low SMI nor low SMD groups.

**Results:**

In total, 443 patients were included, 162 (36.6%) of whom died. Lower SMI and SMD, as continuous variables, were independent risk factors for 90-day mortality (adjusted hazard ratio [HR] = 0.947 and 0.963, respectively, both *p *< 0.001). Cut-off values of L3 SMI and L3 SMD were 32.24 cm^2^/m^2^ and 30.01 HU for men and 28.28 cm^2^/m^2^ and 28.20 HU for women, respectively. The both low SMI and low SMD group had an increased risk of 90-day mortality (adjusted HR=3.059, *p *< 0.001), 90-day readmission (adjusted odds ratio [OR]=2.859, *p *= 0.006), and discharge to long-term care (adjusted OR = 2.814, *p* = 0.007).

**Conclusions:**

Lower muscle mass and muscle quality, as measured by skeletal muscle index and density, were independent risk factors for mortality among older patients with sepsis in the emergency department. Furthermore, patients with both low muscle mass and quality had an increased risk of mortality, readmission, and discharge to long-term care.

## Key summary points

**Aim:**

To explore the association between body composition and adverse clinical outcomes in older patients with sepsis in the emergency department (ED).

**Findings:**

Lower muscle mass and muscle quality were independent risk factors for mortality among older patients with sepsis in the ED. Furthermore, patients with both low muscle mass and quality had an increased risk of mortality, readmission, and discharge to long-term care.

**Message:**

Computed tomography-based body composition may help risk stratification and predict the prognosis for older patients with sepsis.

## Introduction

Older patients (aged ≥65 years) account for 64.9% of all cases of sepsis, a life-threatening organ dysfunction syndrome with a hospital mortality rate of 48.8% [1–3]. The high incidence of sepsis and associated mortality warrants urgent attention to this patient population. Accurate stratification of older patients with sepsis is the process of early identification of critically ill patients requiring emergency care to assist medical decision making and improve prognosis [4].

Sarcopenia is defined as loss of skeletal muscle mass and decreased functional strength [5]. Abdominal computed tomography (CT) is used as a diagnostic examination in the emergency department (ED), and its secondary analysis can be applied to evaluate body composition and define sarcopenia without added economic and radiation burdens to the patient [6]. Previous studies have proved that loss of muscle mass or sarcopenia had an intimate association with mortality of patients with sepsis [7–9].

Previous studies on the association of body composition and prognosis among older patients with sepsis were rare. Shibahashi et al. proved loss of muscle mass was a prognostic risk factor of older patients with sepsis, but muscle quality was not involved [10]. The aim of this study was to evaluate the skeletal muscle area (SMA), skeletal muscle index (SMI), mean skeletal muscle density (SMD), and intramuscular fat area (IFA) using abdominal CT and investigate their association with adverse clinical outcomes in older patients with sepsis in the ED.

## Methods

### Study design and participants

This observational prospective cohort study was conducted at the ED of a tertiary care university-affiliated hospital in Beijing between January 1, 2022, and June 30, 2022. Older patients (≥65 years) with suspected infection admitted to the ED underwent laboratory examination, ultrasound or CT scan. The inclusion criteria were as follows: older patients who met the definition of sepsis-3 [1] and performed abdominal CT scan for diagnosis within 1 week from ED admission. Patients who were discharged or transferred to another hospital within 24 hours of admission and patients whose CT scans did not pass the quality checks (including artifacts, muscle or adipose tissue outside the scanned frame, and poor differentiation between muscle and surrounding tissue) were excluded.

### Demographic and clinical information

The demographic and clinical characteristics included age, sex, body mass index (BMI), presence of cognitive impairment, sites of infection (including lung, abdomen, urinary tract, and others), comorbidities (including hypertension, coronary heart disease, diabetes, stroke, chronic kidney disease, chronic obstructive pulmonary disease, chronic liver disease, solid malignancy, hematological malignancy, and connective tissue disease), Charlson comorbidity index, presence of septic shock, and hospital length of stay (LOS). Clinical frailty scale (CFS) was used for frailty assessment, patients with scores of ≥5 were classified as frail. The worst laboratory indicators within 24 hours after admission were used to calculate the Acute Physiologic and Chronic Health Evaluation (APACHE) II score and sequential organ failure assessment (SOFA) score, as well as other parameters, including lactate level, glucose, albumin, and procalcitonin (PCT).

The patients were followed up for 90 days after ED admission. The primary outcome was all-cause 90-day mortality. Secondary outcomes included 90-day hospital readmission and discharge to long-term care.

### Body composition measured by CT

**Fig. 1:**
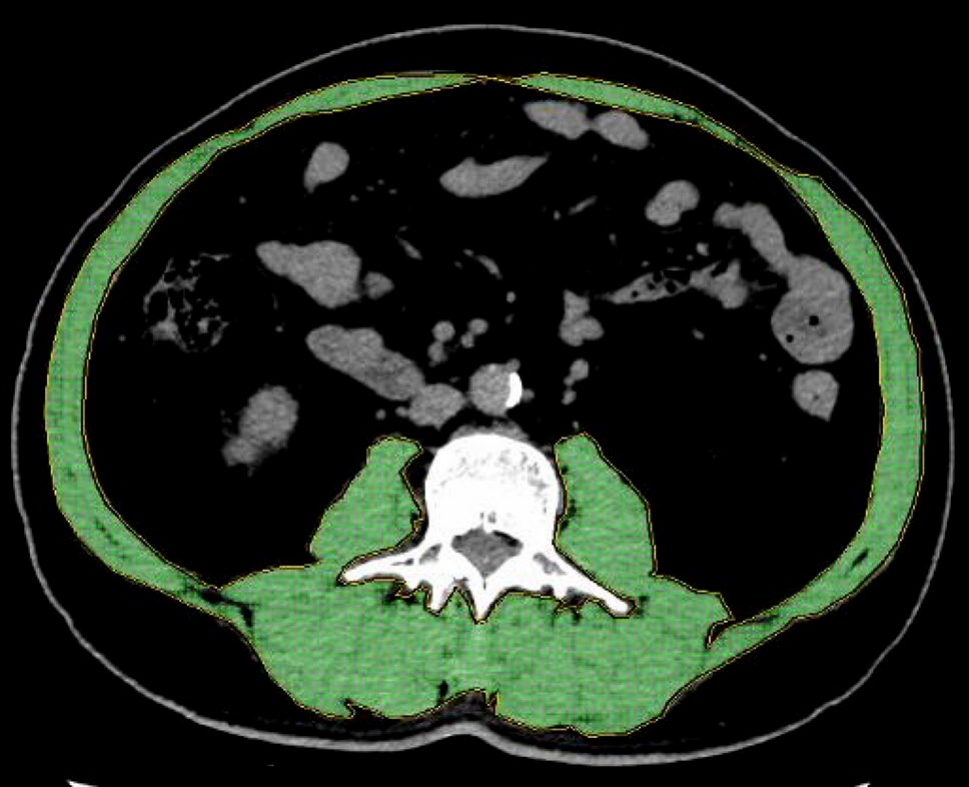
Cross-sectional computed tomography scan at the level of third lumbar vertebra, in which muscle was delineated. The skeletal muscle area (SMA) and mean skeletal muscle density (SMD) can be calculated automatically

CT images were retrieved from the institutional picture archiving and communication system (PACS) and were analyzed using AW Volume Share 7 (GE Medical Systems S.C.S). The CT histogram software "X Section" was used to manually delineate the region of interest (ROI) and automatically calculate the ROI area at the midpoint of the third lumbar vertebra (L3) region of the transverse CT image. The L3 SMA was quantified using Hounsfield unit (HU) thresholds (– 29 to +150) [11]. The SMD was measured by the mean muscle radiation attenuation in HU of all SMA at L3, and it has been shown to be inversely related to muscle lipid content, which is an indicator of muscle quality [12, 13]. The IFA was delineated using fat tissue thresholds (– 190 to – 30 HU) [14], and the SMI was calculated as the SMA (cm^2^) divided by the height squared (m^2^) [6]. All measurements were obtained by one emergency physician who was blinded to the patients' clinical outcomes and had been trained in measuring body composition parameters. (Fig. 1).

### Statistical analysis

Normally distributed variables are expressed as mean (standard deviation [SD]), and they were compared using Student’s *t*-test. Non-normally distributed variables are reported as median (interquartile range [IQR]), and they were compared using the Mann-Whitney U test. Categorical variables are expressed as frequencies (percentages), and they were compared using the chi-square test. Bonferroni correction was used for pairwise comparison. The association between body composition parameters and adverse outcomes was analyzed using Cox proportional hazard and logistic regression models, and variables that were significant in univariable analyses were included in the multivariable analyses. Survival curves were estimated using the Kaplan–Meier method and compared using the log-rank test. A *P*-value of <0.05 was considered significant. Statistical analyses were performed using SPSS version 26.0 (SPSS Inc., Chicago, IL, USA) and GraphPad Prism 9.4.1 Software (GraphPad Software Inc., CA, USA).

We used optimum stratification by X-tile bio-informatics software (version 3.6.1) [15] to determine the most significant p-value via log-rank *x*^2^ statistics and defined the sex-specific cut-off values of L3 SMI and SMD associated with 90-day mortality. This method has been mentioned in previous literature to define the threshold values for continuous variables [6]. These cut-off values were then used to classify patients into four groups as follows: low SMI group, low SMD group, both low SMI and low SMD (both low) group, neither low SMI nor low SMD (neither low) group.

## Results

### Study population

**Fig. 2:**
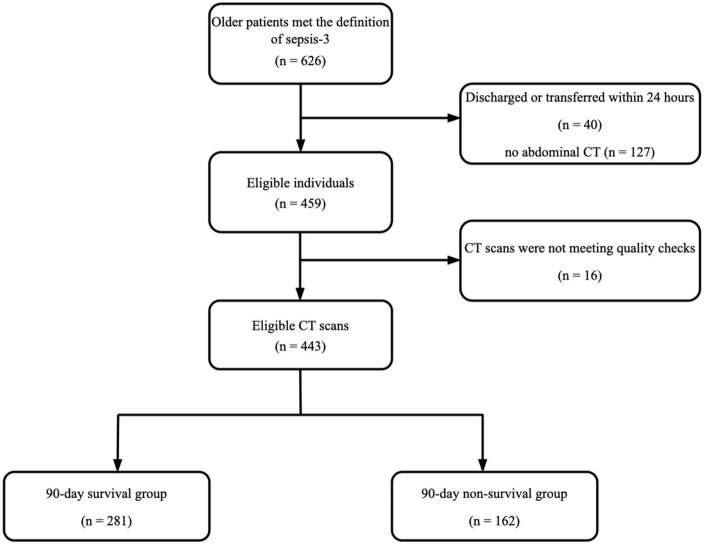
Flowchart of all included and excluded patients

In total, 626 older patients met the definition of sepsis-3 [1]. Patients discharged or transferred within 24 hours (*n* = 40) or without abdominal CT (*n* = 127) were excluded. CT scans of 16 patients did not meet quality checks, including artifacts (7), muscle or adipose tissue outside the scanned frame (5), and poor differentiation between the muscle and surrounding tissue (4). Therefore, 443 patients were finally included and followed up, with a 90-day mortality rate of 36.6% (162 patients died) (Fig. 2). 368 (83.1%) CT scans were performed on admission and 75 (16.9%) were performed within 1 week after admission. There was no significant difference in clinical characteristics between enrolled patients (*n* = 443) and patients without abdominal CT scan (*n* = 127) except for the rate of cognitive impairment (42.0% vs. 22.0%, *p* < 0.001) and the proportion of pneumonia (43.1% vs. 85.0%, *p* < 0.001).

### Comparison of characteristics and body composition according to 90-day mortality

In the overall population, the median age was 79 years (IQR, 16), 244 (55.1%) were male patients, BMI was 22.6 kg/m^2^ (IQR 3.8), 261 (58.9%) were frail patients, and 186 (42.0%) had cognitive impairment. The sites of infection were the lungs (43.1%), abdomen (37.5%), urinary tract (17.6%), and others (1.8%). The etiologies of intra-abdominal sepsis were cholecystitis/cholangitis (56.6%), pancreatitis (24.1%), intra-abdominal abscess (10.8%), and others (8.4%). Compared with survivors, the non-survivors tended to be older (median age, 78 vs. 82 years, *p* = 0.016) and had lower BMI (median, 23.1 vs. 21.8 kg/m^2^, *p* = 0.001), higher prevalence of frailty (46.3% vs. 80.9%, *p* < 0.001), higher rate of solid tumors (29.0% vs. 14.9%, *p* < 0.001), higher Charlson comorbidity index (median, 6 vs. 6, *p* = 0.003), higher APACHE II score (median, 19 vs. 12, *p* < 0.001), higher SOFA score (median, 7 vs. 4, *p* < 0.001), higher rate of septic shock (46.3% vs. 27.4%, *p* < 0.001), higher lactate level (median, 1.8 vs. 1.4 mmol/L, *p* = 0.001), and lower albumin (34.4 vs. 37.0 g/L, *p* < 0.001) (Table 1). The body composition parameters of all patients were stratified by sex and 90-day mortality. Compared with survivors, non-survivors had lower SMA, SMI, and SMD values in overall, male, and female patients (Table 2).

**Table 1:** Comparison of demographic and clinical characteristics between survival and non-survival older patients with sepsis

Characteristics	Overall patients (*n*=443)	Survival group (*n*=281)	Non-survival group (*n*=162)	*P* value
Age (years), median (IQR)	79 (16)	78 (16)	82 (15)	0.016
Male, *n* (%)	244 (55.1)	156 (55.5)	88 (54.3)	0.808
BMI (kg/m^2^), mean (SD)	22.6 (3.8)	23.1 (3.8)	21.8 (3.7)	0.001
Frailty, *n* (%)	261 (58.9)	130 (46.3)	131 (80.9)	< 0.001
Cognitive impairment, n (%)	186 (42.0)	117 (41.6)	69 (42.6)	0.844
Sites of Infection, *n* (%)				0.559
Lung	191 (43.1)	115 (40.9)	76 (46.9)	
Abdomen	166 (37.5)	107 (38.1)	59 (36.4)	
Urinary tract	78 (17.6)	54 (19.2)	24 (14.8)	
Others	8 (1.8)	5 (1.8)	3 (1.9)	
Comorbidities, *n* (%)				
Hypertension	250 (56.4)	152 (54.1)	98 (60.5)	0.191
Coronary heart disease	152 (34.3)	101 (35.9)	51 (31.5)	0.341
Diabetes	166 (37.5)	107 (38.1)	59 (36.4)	0.728
Stroke	135 (30.5)	79 (28.1)	56 (34.6)	0.155
Chronic kidney disease	80 (18.1)	47 (16.7)	33 (20.4)	0.337
Chronic obstructive pulmonary disease	38 (8.6)	27 (9.6)	11 (6.8)	0.308
Chronic liver disease	33 (7.4)	17 (6.0)	16 (9.9)	0.140
Solid malignancy	89 (20.1)	42 (14.9)	47 (29.0)	< 0.001
Hematological malignancy	23 (5.2)	12 (4.3)	11 (6.8)	0.250
Connective tissue disease	14 (3.2)	8 (2.8)	6 (3.7)	0.620
Charlson comorbidity index, median (IQR)	6 (2)	6 (3)	6 (3)	0.003
APACHE II score, median (IQR)	14 (9)	12 (9)	19 (11)	< 0.001
SOFA score, median (IQR)	5 (4)	4 (3)	7 (4)	< 0.001
Septic shock, *n* (%)	152 (34.3)	77 (27.4)	75 (46.3)	< 0.001
LOS, median (IQR)	10 (12)	11 (11)	9 (15)	0.075
Laboratory variables, median (IQR)				
Lactate level (mmol/L)	1.6 (1.5)	1.4 (1.3)	1.8 (1.9)	0.001
Glucose (mmol/L)	8.1 (5.3)	8.0 (4.9)	8.3 (5.6)	0.564
Albumin (g/L)	36.1 (8.3)	37.0 (7.3)	34.4 (8.0)	< 0.001
PCT (ng/mL)	1.0 (6.8)	0.8 (7.7)	1.2 (5.2)	0.248

*IQR* Interquartile Range, *BMI* body mass index, *SD *standard deviation, *APACHE II score* Acute Physiology and Chronic Health Evaluation II score, *SOFA* score Sequential Organ Failure Assessment score, *LOS* length of stay, *PCT* procalcitonin

**Table 2:** Comparison of body composition parameters between survival and non-survival older patients with sepsis by sex

Body Composition	Overall			Male			Female		
	Survival group (*n*=281)	Non-survival group (*n*=162)	*P* value	Survival group (*n*=156)	Non-survival group (*n*=88)	*P* value	Survival group (*n*=125)	Non-survival group (*n*=74)	*P* value
SMA (cm^2^), mean (SD)	96.86 (24.72)	81.83 (22.62)	<0.001	107.50 (23.66)	91.61 (22.41)	<0.001	83.58 (18.97)	70.20 (16.64)	< 0.001
SMI (cm^2^/m^2^), mean (SD)	35.39 (8.05)	29.93 (7.55)	<0.001	37.02 (8.40)	31.45 (7.61)	<0.001	33.35 (7.12)	28.12 (7.12)	< 0.001
SMD (HU), mean (SD)	27.00 (9.72)	21.88 (9.03)	<0.001	31.04 (8.50)	25.05 (8.74)	<0.001	21.95 (8.76)	18.10 (7.89)	0.002
IFA (cm^2^), median (IQR)	18.98 (14.71)	16.91 (16.71)	0.021	17.97 (13.84)	15.82 (13.08)	0.083	20.12 (15.59)	18.96 (17.93)	0.129

*SMA* skeletal muscle area, *SD* standard deviation, *SMI* skeletal muscle index, *SMD* mean skeletal muscle density, *HU* Hounsfield units, *IFA* intramuscular fat area, *IQR* interquartile range

### Association between body composition and 90-day mortality

The univariable Cox proportional hazard model showed that lower SMI, SMD, and IFA, as continuous variables, were risk factors for 90-day mortality (hazard ratio [HR] = 0.931, *p* < 0.001; HR = 0.960, *p* < 0.001; and HR = 0.984, p = 0.020, respectively). After adjusting for age, sex, BMI, frailty, Charlson comorbidity index, APACHE II score, SOFA score, presence of septic shock, lactate level, and albumin, lower SMI and SMD were independent risk factors for 90-day mortality (adjusted HR = 0.947 and 0.963, respectively, both *p* < 0.001) (Table 3).

**Table 3:** Results of Cox proportional hazard models for 90-day mortality

Body composition parameters	Univariable analysis		Multivariable analysis	
	HR (95% CI)	*P* value	HR (95% CI)	*P* value
Continuous variables				
SMI (per 1 cm^2^/m^2^ increase)	0.931 (0.912–0.951)	< 0.001	0.947 (0.924–0.970)	< 0.001
SMD (per 1 HU increase)	0.960 (0.945–0.976)	< 0.001	0.963 (0.946–0.980)	< 0.001
IFA (per 1 cm^2^ increase)	0.984 (0.970–0.997)	0.020	−	−
Dichotomous variables by cut-off values				
Neither low SMI nor low SMD (neither low) group	1[reference]	NA	1[reference]	NA
Low SMI group	2.398 (1.104–5.208)	0.027	1.752 (0.785–3.908)	0.171
Low SMD group	1.912 (1.156–3.162)	0.012	1.692 (0.989–2.894)	0.055
Both low SMI and low SMD (both low) group	4.475 (2.792–7.172)	< 0.001	3.059 (1.827–5.121)	< 0.001

Adjusted for age, sex, BMI, frailty, Charlson Comorbidity Index, APACHE II score, SOFA score, presence of septic shock, lactate level, albumin

*SMI* skeletal muscle index, *SMD* mean skeletal muscle density, *HU*, Hounsfield units, *IFA* intramuscular fat area, *HR*, hazard ratio, *CI* confidential interval, *BMI* body mass index, APACHE II score, Acute Physiology and Chronic Health Evaluation II score, *SOFA* score, Sequential Organ Failure Assessment score

We used optimum stratification to determine the threshold values for continuous variables, which can best separate patients concerning the time-to-an-event outcome (mortality). The sex-specific cut-off values of L3 SMI and L3 SMD were 32.24 cm^2^/m^2^ and 30.01 HU for men and 28.28 cm^2^/m^2^ and 28.20 HU for women, respectively. Patients with low SMI and SMD had lower values than their respective cut-off values.

**Fig. 3:**
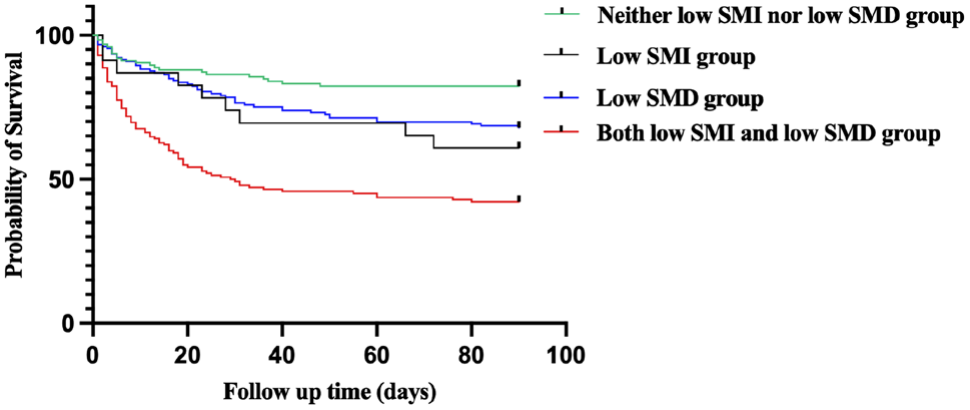
Kaplan-Meier survival curves of 90-day mortality for the four groups

Therefore, the patients were divided into four groups: 23 (5.2%) in the low SMI group, 153 (34.5%) in the low SMD group, 142 (32.1%) in the both low group, and 125 (28.2%) in the neither low group. The 90-day mortality rates were significantly varied among the four groups (*p* < 0.001). Pairwise comparisons revealed that the both low group was significantly different from the neither low group and the low SMD group (57.7% vs. 17.6%, 57.7% vs. 32.0%, respectively; both *p* < 0.001). The survival curves also corroborated the differences among the four groups (log-rank: *x*^2^ = 55.19, *p* < 0.001) with significant differences between the neither low group and the both low group (log-rank: *x*^2^ = 44.85, *p* < 0.001) and between the low SMD group and the both low group (log-rank: *x*^2^ = 24.04, *p* < 0.001) (Fig. 3). The univariable Cox proportional hazard model showed that compared with those in the neither low group, patients in the low SMI group, low SMD group, and both low group had higher risks of 90-day death (HR = 2.398, 1.912, and 4.475; *p* = 0.027, *p* = 0.012, and *p* < 0.001, respectively). Further analysis adjusted for age, sex, BMI, frailty and other potential confounders showed that the both low group had an increased risk of 90-day mortality (adjusted HR = 3.059, *p* < 0.001) (Table 3).

### Association between body composition and secondary outcomes

Patients who survived until discharge were analyzed for 90-day readmission and discharge to long-term care. In total, 309 patients were divided into four groups: 17 (5.5%) in the low SMI group, 112 (36.2%) in the low SMD group, 75 (24.3%) in the both low group, and 105 (34.0%) in the neither low group. The rates of 90-day readmission and discharge to long-term care were significantly different among the four groups (both *p* < 0.001). Pairwise comparisons revealed significant differences in the rates of readmission (52.0% vs. 20.0%, respectively; *p* < 0.001) and discharge to long-term care (57.3% vs. 21.0%, respectively; *p* < 0.001) between the both low group and the neither low group. The rate of discharge to long-term care was also significantly different between the both low group and the low SMD group (57.3% vs. 33.0%, respectively; *p* < 0.001). The univariable logistic regression models showed that compared with those in the neither low group, patients in the low SMI group, low SMD group, and both low group had higher likelihoods of 90-day readmission (OR = 3.556, *p* = 0.020; OR = 2.222, *p* = 0.011; and OR = 4.333, *p* < 0.001, respectively) and discharge to long-term care (OR = 3.354, *p* = 0.026; OR = 1.861, *p* = 0.047; and OR = 5.070, *p* < 0.001, respectively). After adjusting for confounders, the both low group had an increased risk of 90-day readmission (adjusted OR = 2.859, *p* = 0.006) as well as discharge to long-term care (adjusted OR = 2.814, *p* = 0.007).

## Discussion

Using a secondary analysis of abdominal CT, we assessed body composition and its impact on survival among older patients with sepsis in the ED. We defined the cut-off values for SMI and SMD associated with survival and grouped them accordingly. Furthermore, we analyzed the impact of low SMI and low SMD on the rates of 90-day readmission and discharge to long-term care. To our knowledge, no study to date has assessed the association between body composition and adverse clinical outcomes among older patients with sepsis in the ED in China.

In this study, the mortality rate of older patients with sepsis was 36.6%, which approximated that reported in a previous study (38.7%) [10]. In consensus with recent meta-analyses [7, 8], we found that lower muscle mass, as a continuous variable, was an independent risk factor for 90-day mortality. However, another study showed that muscle wasting–associated comorbidities, rather than sarcopenia, were risk factors for hospital mortality in critically ill patients with abdominal sepsis [16]. Population heterogeneity and the different cut-off values for defining sarcopenia may account for these differences.

The primary strength of this study is that skeletal muscle mass was considered as well as muscle quality indicators, including SMD and intramuscular adipose tissue [17]. We found that unlike intramuscular fat area, lower SMD was the independent risk factor for 90-day mortality. Though this phenomenon has been verified in patients with cirrhosis or ovarian cancer [18, 19], no study has reported an association between muscle quality and prognosis in older patients with sepsis in the ED.

CT is a standard method for non-invasive measurement of muscle mass [20]. Though CT-based muscle measurement (L3 SMA) is significantly correlated with whole-body muscle mass [6], the cut-off values for low muscle mass or sarcopenia are not uniform. Van der Werf et al. have reported sex-specific percentiles for L3 SMI in a healthy Caucasian population and thresholds to define sarcopenia (41.6 cm^2^/m^2^ and 32.0 cm^2^/m^2^ for men and women, respectively) [21]. Some investigators determined cut-off values using receiver operating characteristic (ROC) curve analysis [10, 22]. Prado et al. defined cut-off values of 52.4 cm^2^/m^2^ and 38.5 cm^2^/m^2^ for men and women, respectively, among obese patients with solid tumors of the respiratory and gastrointestinal tracts [6]. A study in China defined the cut-off values of 40.8 cm^2^/m^2^ and 34.9 cm^2^/m^2^ for men and women, respectively, among patients who underwent radical gastrectomy for gastric cancer [23]. Like previous studies [6, 23], we also used optimal stratification analysis for survival to define cut-off values of L3 SMI. Our cut-off values were 32.24 cm^2^/m^2^ and 28.28 cm^2^/m^2^ for men and women, respectively, which were lower than those reported earlier, likely because our study population comprised adults aged ≥65 years. Another possible reason for this discrepancy could include race, as our study was conducted in an Asian country. Additionally, we also defined cut-off values of L3 SMD, which were 30.01 HU and 28.20 HU in men and women, respectively. However, the threshold values were higher than those reported in a previous study (29.3 HU and 22.0 HU in men and women) [21]. The primary reason for this variation may be the different methods of defining cut-off values.

Furthermore, the association between body composition and secondary outcomes was also analyzed. We considered potential confounders and found that patients with both low SMI and SMD had increased likelihoods of 90-day readmission and discharge to long-term care. We performed subgroup analyses to gain insight into the separate impacts of SMI and SMD on prognosis. Pairwise comparisons revealed that the both low group had a higher mortality rate and higher rate of discharge to long-term care than the neither low group and the low SMD group. This may indicate that low muscle mass has a greater influence on adverse clinical outcomes in older patients with sepsis.

This study has several limitations. First, this was a single-center study, and patients were included according to the availability of CT scans, thus potentially contributing to selection bias. Second, Older patients generally suffer from acute muscle wasting during hospitalization, both sarcopenia and acute muscle wasting have potentially negative clinical outcomes. This study was based on an analysis of single-occurrence CT scans, and changes in body composition were not assessed dynamically. Third, body composition did not include subcutaneous and visceral adipose tissues. A previous study has reported that the increased ratio of visceral to subcutaneous adipose tissue was associated with adverse outcomes in patients with sepsis [24]. Fourth, low muscle strength is an important part of the sarcopenia diagnosis [25]; however, we did not consider it because patients in ED, especially those who are acutely, critically ill, cannot complete the physical function examination.

In conclusion, this study analyzed the association between body composition and adverse clinical outcomes among older patients with sepsis in an ED in China. We investigated that lower muscle mass and quality, as measured by SMI and SMD, respectively, were independent risk factors for mortality. Furthermore, patients with both low muscle mass and quality had an increased risk of mortality, readmission, and discharge to long-term care. Our findings suggest that clinicians pay attention to assessment of body composition as well as diagnostic images. Identifying patients with low muscle mass and quality may contribute to better stratification of older patients with sepsis.

## Data Availability

The data generated and/or analyzed during this study is available from the corresponding author upon reasonable request.
